# METTL3-Mediated m^6^A RNA Methylation of ZBTB4 Interferes With Trophoblast Invasion and Maybe Involved in RSA

**DOI:** 10.3389/fcell.2022.894810

**Published:** 2022-06-14

**Authors:** Nana Huang, Yue Gao, Mengting Zhang, Liangjie Guo, Litao Qin, Shixiu Liao, Hongdan Wang

**Affiliations:** ^1^ Henan Provincial People’s Hospital, Medical Genetics Institute of Henan Province, Zhengzhou University People’s Hospital, Zhengzhou, China; ^2^ National Health Commission Key Laboratory of Birth Defects Prevention, Henan Key Laboratory of Population Defects Prevention, Henan Institute of Reproduction Health Science and Technology, Zhengzhou, China

**Keywords:** N^6^-methyladenosine, METTL3, ZBTB4, recurrent spontaneous abortion, trophoblast invasion

## Abstract

N^6^-methyladenosine (m^6^A) was the most abundant modification of mRNA and lncRNA in mammalian cells and played an important role in many biological processes. However, whether m^6^A modification was associated with recurrent spontaneous abortion (RSA) and its roles were still unclear.

**Methods:** Methylated RNA immunoprecipitation sequencing (MeRIP-Seq) was used to study the global m^6^A modification pattern in RSAs and controls. RNA sequencing (RNA-Seq) was used to study the level of global mRNA in two groups. Real-time quantitative PCR (RT-qPCR) was used to verify the level of mRNA of METTL3 and ZBTB4. MeRIP–qPCR was conducted to test the level of ZBTB4 m^6^A modification in two groups. In order to further explore whether ZBTB4 was the substrate of METTL3, the HTR-8/SVneo (HTR-8) cell line was selected for the knockdown and overexpression of METTL3. To study whether METTL3 regulated the ZBTB4 expression by recognizing ZBTB4 mRNA m^6^A motifs in coding sequences (CDS), dual-luciferase reporter assay was conducted. RNA stability assays using actinomycin D were conducted to study the RNA stability of the HTR-8 cell line with METTL3 overexpression and knockdown. To illustrate the role of METTL3 in the invasion of trophoblast, matrigel invasion assays and transwell migration assays were conducted using the HTR-8 cell line with METTL3 overexpression and knockdown.

**Results:** A total of 65 genes were found with significant differences both in m^6^A modification and mRNA expression. We found m^6^A methyltransferase METTL3 was significantly down-regulated in the RSA group. Through gene function analysis, RT-qPCR, MeRIP–qPCR validation experiment, knockdown, and overexpression of METTL3 in the HTR-8 cell line, ZBTB4 was selected as one target of METTL3. Furthermore, we clarified that METTL3 regulated the expression of ZBTB4 by recognizing ZBTB4 mRNA m^6^A motifs in the CDS using the dual-luciferase reporter assay and METTL3 regulated the invasion of trophoblast by altering the stability and expression of ZBTB4 by RNA stability, matrigel invasion, and transwell migration assays.

**Conclusion:** Our study revealed the mechanism by which METTL3 regulated the stability and expression of ZBTB4 and the trophoblast migration ability of RSA. A new perspective was provided for exploring the mechanism of embryonic development in RSA patients.

## Introduction

As one of epigenetic regulation, N^6^-methyladenosine (m^6^A) methylation is a new field. M^6^A is the most abundant modification of mRNA and lncRNA in mammalian cells and plays an important role in cell growth and differentiation (Roundtree et al., 2017). M^6^A modification can regulate mRNA stability, selective shearing, transport, and translation at the post-transcriptional level, thus affecting various biological processes, such as embryonic development, proliferation and migration of cancer cells, and self-renewal of stem cells (Zheng et al., 2013; Mishima and Tomari, 2016; Li et al., 2017; Liu et al., 2017; Huang et al., 2018). From catalytic formation to functional realization, m^6^A methylation is mainly regulated by the interaction of m^6^A methyltransferase complexes such as methyltransferase-like 3 and 14 (METTL3 and METTL14), m^6^A demethylases such as fat mass and obesity-associated protein and AlkB homolog 5 (FTO and ALKBH5), and m^6^A-binding proteins such as YT521-B homology domain family 1/2/3(YTHDF1/2/3) which is a dynamic modification of RNA (Yang et al., 2018). Among them, METTL3 has been shown to regulate embryo development (Bodi et al., 2012; Wang et al., 2018), spermatogenesis (Xu et al., 2017), and cell reprogramming (Aguilo and Walsh, 2017). Zebrafish embryos with WTAP and METTL3 knockout showed a variety of developmental defects, suggesting that WTAP and METTL3 could affect embryonic tissue differentiation (Ping et al., 2014). In both studies, the inactivation of METTL3 in mice or *Drosophila* resulted in the failure of embryo development, suggesting that m^6^A modifications may be involved in embryo differentiation and development (Geula et al., 2015; Meng et al., 2019). In germ cells, the deletion of METTL3 severely inhibits sperm differentiation and meiosis (Xu et al., 2017).

Recurrent spontaneous abortion (RSA) is the most common complication of pregnancy. Pregnancy failure in early pregnancy seriously affects the reproductive health. Unexplained recurrent spontaneous abortion (URSA) accounts for 50% of RSA (Arias-Sosa et al., 2018). Severe placental insufficiency is the pathogenic factor of many implant failures and early abortions. The migration and invasion of trophoblast cells at the maternal–fetal interface is a key process of embryo implantation. Inadequate invasion of trophoblast cells may cause abortion, fetal growth restriction, fetal death, preeclampsia, etc. (Kaufmann et al., 2003; Soares et al., 2014). Li et al. mapped the m^6^A modification profile of preeclampsia placenta and found that METTL3 and METTL14 were up-regulated in preeclampsia. Differential m^6^A-modified transcripts are primarily associated with the nitrogen compound metabolic process, positive regulation of vascular-associated smooth muscle cell migration, and several cancer-related pathways (Wang et al., 2020a). Li et al. also found that the reduced methylation of m^6^A in RSA villous may inhibit the invasion of villous cells and lead to RSA (Wang et al., 2020a). However, whether m^6^A modification can lead to RSA and its exact mechanisms still need to be proved by a large number of studies.

Zinc finger and BTB domain containing 4 (ZBTB4), a member of ZBTB4 protein family, functions as a transcriptional repressor (Roussel-Gervais et al., 2017). Previous studies reported that ZBTB4 had cancer suppressive effects in humans and the expression was decreased in various cancers, including breast cancer, lung cancer, gastric cancer, and so on (Kim et al., 2012; Fan et al., 2019; Cheng et al., 2021). METTL3 is highly expressed in the lung tissues of smokers and negatively correlated with ZBTB4. M^6^A modification of ZBTB4 mediated by EZH2 is involved in CS-induced epithelial–mesenchymal transition (EMT) and lung cancer (Kim et al., 2012). These studies suggest that the alteration of ZBTB4 may affect the differentiation and proliferation migration of tissue cells, leading to the transformation of cancer. However, whether the proliferation and migration of villous trophoblast cells in RSA are affected by ZBTB4 remain to be explored.

In this study, we analyzed the m^6^A methylation modification mode and differential modification transcription in RSA villous tissues and controls by MeRIP-seq and RNA-seq, and found that the expression of m^6^A methyltransferase METTL3 in chorionic villous tissues of RSA patients was significantly lower than that of normal controls. The expression of ZBTB4 was significantly increased. Further experiments showed that METTL3 up-regulated the expression of ZBTB4 by mediating m^6^A modification, and thus weakened the proliferation and migration of trophoblast cells in RSA villous tissue. Our research provided a new perspective for exploring the mechanism of embryonic development in RSA patients.

## Methods and Materials

### Patients and Samples

Recurrent spontaneous abortion patients (RSA group) were recruited from the patients with multiple embryo abortions for unexplained reasons and normal control pregnancy samples (NC group) from the persons who voluntarily terminated their pregnancy in People’s Hospital of Zhengzhou University from June 2019 to August 2020. The inclusion criteria of the RSA group included the following: spontaneous abortion ≥2 times before 10 weeks of gestation, last spontaneous abortion within 1 week, intrauterine pregnancy indicated by ultrasonography, gestational sac stopped developing (or less than normal gestational age), gestational sac without the heartbeat, and empty gestational sac. The inclusion criteria of the NC group included the following: ultrasound suggested intrauterine pregnancy, gestational age less than 10 weeks, at least one normal birth history, and no adverse pregnancy and birth history. Chromosome abnormality and common etiology of RSA were excluded in both groups. Embryonic villous tissues were collected from both groups.

This study has been reviewed by the Ethics Committee of the People’s Hospital of Zhengzhou University. All the patients participating in this study have signed their informed consents.

### m^6^A Methylated RNA Immunoprecipitation–Seq Assay and Data Analysis

Total RNA of villous tissues in both groups was extracted using the TRIzol Reagent (Life technologies, MA, United States) and was examined using NanoDrop-2000 (Thermo Fisher Scientific, MA, United States). MeRIP-Seq was carried out referring to the procedure of the published reference (Li et al., 2019). First, the RNA samples needed to be ultrasonically interrupted as fragmentations (∼100 nt) and the RNA fragment reagent (Millipore, MA, United States) was used. Second, the fragmented RNA samples were divided into two parts: the input control group and immunoprecipitation (IP) group. The IP group was then enriched using EpiMark^®^ N6-Methylation adenine Enrichment Kit (NEB, MA, United States). The final steps performed were the synthesis of double-stranded cDNA by reverse transcription, random primed cDNA library generation, adaptor ligation, and Illumina sequencing. The following kits were used to complete the final steps: Ribo-Zero Magnetic Gold Kit (Illumina, MA, United States) and NEB Next Ultra RNA Library Prep Kit (NEB, MA, United States). The next-generation sequencing was performed on m^6^A IP and input samples were obtained using the Illumina HiSeq platform (Illumina, MA, United States).

The raw data of each sample was trimmed using the fastp software version 0.20.0 to get the high-quality clean reads (>50 bp). Bowtie2 version 2.2.5 software was used to align the high-quality clean reads to the human genome hg19 reference. Only the reads aligned to the unique position on the reference genome were used for subsequent analysis. ExomePeak2 version 1.0.0 was used to scan the peaks of m^6^A in the whole genome and identify the sequence information of the region where the peaks were located, the positions on the genome, and the related genes. Then, the methylation rate of each peak of the input control group and IP group was calculated. Using the input data as a control, the exomePeak2 software was used to analyze the difference in m^6^A RNA methylation rate of all peaks between the two groups, and the peaks with significant differences were screened under the conditions of *p* < 0.05 and |log2FC| > 1. The MEME Suite software was used to identify the specific binding sequences (the motifs) in the m6A peaks enriched in. The Gene Ontology (GO) analysis and Kyoto Encyclopedia of Genes and Genomes (KEGG) analysis were also conducted using the ClusterProfile package of R software.

### mRNA Sequencing

Total RNA was collected using a TRIzol reagent (Life technologies, MA, United States). RNA’s purity and concentration were defined by OD260/280 using a spectrophotometer NanoDrop-2000 (Thermo Fisher Scientific, MA, United States). RNA integrity was defined by 1% formaldehyde denaturing gel electrophoresis. RNA integrity was defined by capillary electrophoresis using Bioanalyzer 2,100 (Agilent Technologies, CA, United States). Library construction was conducted using the NEBNext^®^ Ultra™ RNA Library Prep Kit for Illumina^®^ Kit (NEB, MA, United States) and then sequenced on the Illumina HiSeq system (Illumina, MA, United States). Disease Ontology (DO), GO, and KEGG analyses were conducted for the target genes of significant difference.

### Quantitative Real-Time PCR

The RNA was extracted using the TRIzol method, which was reverse transcribed to cDNA using a Thermo First Strand cDNA Synthesis Kit (Thermo Fisher Scientific, MA, United States) and HiScript Reverse Transcriptase Kit (Vazyme Biotech Co., Nanjing, China). Reaction amplification was performed using the ABI StepOne Real-Time PCR system (ABI, Vernon, United States). The reaction conditions were as follows: 95°C, 10 min; 95°C, 20 s and 60°C, 60 s for a total of 40 cycles. The primers used for RT-qPCR were shown in Table 1.

**TABLE 1:** Primer sequences for RT-qPCR.

Gene	Primer sequence (5′-3′)	The size of PCR product
METTL3-1	F:GATGCGCAGGCTCAACATAC	224bp
	R:TGGAACGAACCAAGCAGTGT	
METTL3-2	F: AAC​CTC​TGG​GGG​TAT​GAA​CGG​GTA	151bp
	R: TGA​AGC​CTT​GGG​GAT​TTC​CTT​TGA​C	
ZBTB4-1	F:AGGAAGTACCCCTGCCGCTA	215bp
	R:TTGTAGCCTCCATTGGGTGT	
MTR	F:GAGCCCTTCAGGATTGGACC	148bp
	R:CTCCCATTTCCACCTGCACT	
EMILIN1	F:GGCAACCAAGGACCGTATCA	172bp
	R:CCGTTCACAGACACCCTCAA	
LGI2	F:TGACGTGACCAGCTTTGACT	218bp
	R:CCACGATGGACTGACCTGTA	
TGFA	F:CTCCTGAAGGGAAGAACCGC	119bp
	R:TGCTGTCCTGAAGAAGCCTTT	
PLSCR3	F: ATC​CCT​TCC​TCC​CCA​AGT​TCT	239bp
	R: CTT​CAC​CCT​CAC​ATC​CAG​GTC	
β-Actin	F: GTG​GCC​GAG​GAC​TTT​GAT​TG	73bp
	R: CCT​GTA​ACA​ACG​CAT​CTC​ATA​TT	
GAPDH	F: TCA​AGA​AGG​TGG​TGA​AGC​AGG	115bp
	R: TCA​AAG​GTG​GAG​GAG​TGG​GT	

### Cell Culture, Transfection, and siRNAs for METTL3

The HTR-8/SVneo cell line was used for the follow-up assay purchased from Procell Co. (Procell Co., Wuhan, China) and was cultured in a DMEM medium (Gibco, Thermo Fisher) containing 10% fetal bovine serum at 37°C and 5% CO_2_. We constructed the PLVX-mCMV-ZsGreen-IRES-Puro-METTL3 plasmid and the control vector for the overexpression of METTL3 purchased from JTS scientific (JTS scientific Co., Wuhan, China). Lipofectamine 2000 (Invitrogen, CA, United States) was used for the cell transfection. The METTL3-small interfering RNAs (siRNAs) and the negative control siRNA were purchased from JTS scientific (JTS scientific Co., Wuhan, China). METTL3 knockdown was conducted using specific siRNA. In order to select the siRNA with the best interference effect, we designed three siRNAs. METTL3 siRNA #1 sequence: 5′-GCA​AGA​AUU​CUG​UGA​CUA​UTT-3’; METTL3 siRNA #2 sequence: 5′-GGA​UAC​CUG​CAA​GUA​UGU​UTT-3’; METTL3 siRNA #3 sequence: 5′-GCU​ACC​UGG​ACG​UCA​GUA​UTT-3’. The final transfected concentration of the oligonucleotides was 100 mM/L and Lipofectamine 2000 and Opti-MEM^®^ (Thermo Fisher Scientific, MA, United States) were used.

### ELISA Assay for RNA m^6^A Quantification

The quantification of total RNA m^6^A modification was carried out in the HTR-8/SVneo cell line with METTL3 overexpression and knockdown using the ELISA assay to understand the changing of total m^6^A modification after METTL3 overexpression and knockdown. The EpiQuil m^6^A RNA Methylation Quantification Kit (Colorimetric) (Epigentek Group Inc., New York, United States) and Flexstation^®^ 3 (Molecular Devices, CA, United States) were used for RNA m^6^A quantification. The experiment operation was carried out in accordance with the instructions of the kits.

### MeRIP–qPCR

The RNA-Binding Protein Immunoprecipitation Kit (Millipore, MA, United States) was used for MeRIP–qPCR and the assay was conducted according to the protocol of the kit. The N6-methyladenosine (m^6^A) polyclonal antibody needed for the assay was purchased from Epigentek (Epigentek Group Inc., New York, United States). The primer used for MeRIP-qPCR was as follows: ZBTB4 forward: 5′- TTA​GGC​TTG​TAG​GAG​TAG​TAG​GG-3′, ZBTB4 reverse: 5′- TGT​GTC​AGA​TCA​CTG​TGC​GAA​TAG-3’. The reaction conditions were the same as RT-qPCR.

### Western Blotting

Western blotting (WB) was carried out according to the method of our laboratory (Wang et al., 2020b). RIPA lysate was used to extract the total protein from the cell line purchased from Beyotime (Beyotime Biotechnology, Shanghai, China). Antibodies used for WB were diluted according to the instructions of the anti-METTL3 antibody (Affinity BioReagents, Melbourne, United States), anti-ZBTB4 antibody (Abcam, Cambridge, United Kingdom), anti-GAPDH (Abcam, Cambridge, United Kingdom), and HRP conjugated AffiniPure goat anti-rabbit secondary antibody (Boster Biological Technology co., Wuhan, China).

### Dual-Luciferase Reporter Assay

ZBTB4 CDS containing m^6^A motifs (wild-type and mutant) was inserted into the pGL3-Basic psiCheck2 vector which was a gift from Wuhan Botao Biotechnology Co.. The insert fragment of ZBTB4 CDS contained the region m^6^A peaks enriched. For the dual-luciferase reporter assay, the cells were processed in two different ways: ⅰ, cotransfect ZBTB4 CDS pGL3-Basic psiCheck2 vector and overexpression METTL3 plasmid, or ⅱ, control. After 24 h of processing, the activities of firefly luciferase and Renilla luciferase were detected using the Dual-Luciferase Reporter Gene Assay Kit (Beyotime Biotechnology, Shanghai, China) and the operation was carried out according to the protocol.

### RNA Stability Assays

Actinomycin D, an autophagy activator, was used to study the RNA stability of the HTR-8/SVneo cell line with METTL3 overexpression and knockdown purchased from MCE (MCE, NJ, United States). Actinomycin D was added to the HTR-8/SVneo cell line treated with overexpression plasmid or siRNA for 24 h. The final concentration of actinomycin D we used at every time point was 5 mg/ml. The HTR-8/SVneo cell line was collected at the three time points of 0, 2, and 4 h for RNA extraction, reverse transcription, and qPCR, respectively. We got the relative ZBTB4 mRNA levels at the three time points of 0, 2, and 4 h and plotted a line chart.

### Immunofluorescence

HTR-8 cells were transfected with METTL3 siRNA oligonucleotide (#1) or with the METTL3 overexpressing vector and cultured at a density of 2.5×10^4^ cells/well on coverslips placed on the wells of 24-well plates at 37°C for 24 h. Subsequently, the cells were treated as follows: 4% paraformaldehyde for 15 min, permeabilization with 1% Triton X-100 for 5 min, 5% goat serum for 30 min, ZBTB4 antibody (Abcam, Cambridge, United Kingdom) for 1:250 dilution treatment in the 5% goat serum blocking solution at 4 °C overnight, secondary antibody treatment (1:400 dilution) in the dark at room temperature for 1 h, washed three times with PBS, and placed the coverslips onto the microscope slides with DAPI (Thermo Fisher Scientific, MA, United States). Fluorescent signals were photographed using a confocal laser-scanning microscope (Olympus Corporation, Tokyo, Japan).

### Matrigel Invasion and Wound Healing Assays

HTR-8 cells were transfected with METTL3 siRNA oligonucleotide (#1) and METTL3 overexpressing vector separately. The transfected cells that were in the logarithmic growth phase grew well, inoculated 2×10^5^ cells per well in a 6-well plate, and cultivated overnight in an incubator at 37°C and 5% CO_2_ before matrigel invasion assays. Four groups were set up, namely, the overexpressing control group (OE-NC), overexpressing group (OE-METTL3), METTL3 knockdown group (siMETTL3#1), and knockdown control group (siCtrl). Matrigel was purchased from Corning BioCoat (Corning^®^ Biocoat, MA, United States). The transwell was purchased from BD Biosciences (BD, NJ, United States).

The aforementioned HTR-8 cells were washed twice with PBS and resuspended in a serum-free 1,640 medium at a density of 2 × 10^4^cells/ml. Matrigel was diluted with a serum-free medium to the final concentration of 1 mg/ml. The bottom of the transwell (24-well plate) was supplied with a 800 μl 1,640 medium containing 10% FBS. The transwell was added to the 100 μl matrigel solution and incubated at 37°C for 4–5 h. After matrigel was dried into a gel, 200 μl of the cell suspensions of each group were inoculated into the upper chamber of the transwell and cultivated in an incubator at 37°C and 5% CO_2_ for 24 h. Then, the transwell was taken out, washed with PBS, and the cells were fixed with 70% ice ethanol solution for 1 h and stained with 0.5% crystal violet for 20 min at room temperature. The migrated cells were, then, photographed using a inverted microscope (MSHOT, Guangzhou, China).

The aforementioned HTR-8 cells (four groups) inoculated in a 6-well plate were wounded with a sterile scratcher. The cell migration was determined by measuring the changes in the area of the wounds using the inverted microscope (MSHOT, Guangzhou, China).

## Results

### The m^6^A Modification Pattern in RSA and Control Trophoblast Cells

**FIGURE 1:**
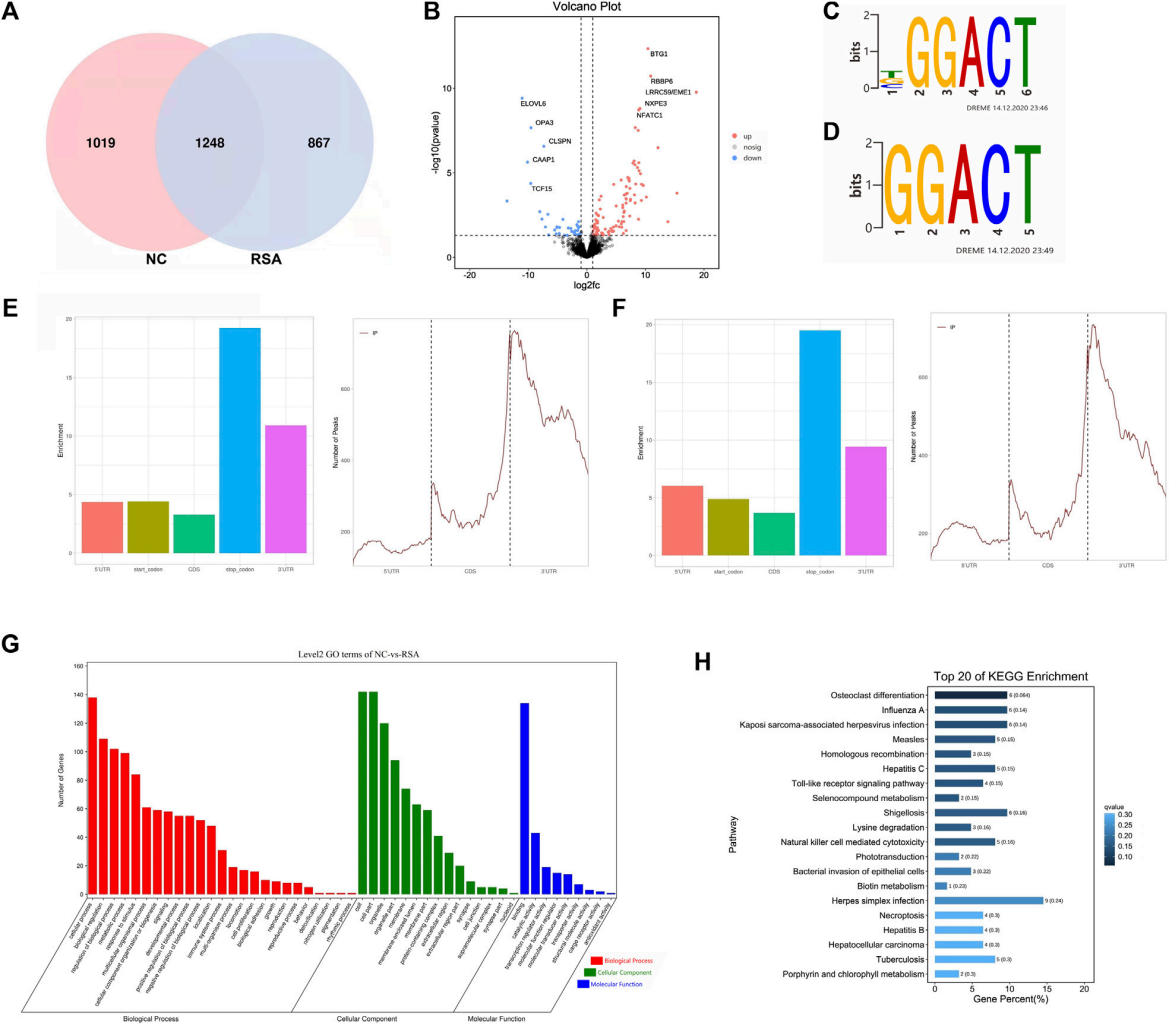
Overview of m^6^A modification pattern in the RSAs and controls. **(A)** Venn diagram of the m^6^A modification peaks in villous tissues of RSA and control groups showing the overlapping m^6^A modification peaks of the two groups. **(B)** Volcano plot showed that there were 101 hyper-methylated and 37 hypo-methylated m^6^A peaks (fold change >2, *p* < 0.05) in RSAs compared with controls. **(C,D)** Top m^6^A motifs enriched from all identified m^6^A peaks in RSA and control groups. **(E)** Distribution of m^6^A modification peaks in the villous mRNA transcriptome of the control group. **(F)** Distribution of m^6^A modification peaks in the villous mRNA transcriptome of the RSA group. **(G)** Gene Ontology (GO) analysis was performed on genes with significantly different m^6^A modification between the two groups. **(H)** Kyoto Encyclopedia of Genes and Genomes (KEGG) analysis performed on genes with significantly different m^6^A modification between the two groups and the top 20 are shown.

In order to study the m^6^A modification pattern in RSA patients, we conducted the MeRIP-Seq in six RSA patients (RSA group) and normal controls (NC group). After quality control and analysis, a total of 2,115 m^6^A peaks associated with 2,883 genes were identified in the RSA group. Also, a total of 2,267 m^6^A peaks associated with 2,669 genes were identified in the NC group. According to the results, we draw the Venn diagram (Figure 1A). We also found that the highest m^6^A modification peaks were present on chromosome 17, followed by chromosomes 1, 2, 12, and 19 (Supplementary Figure S1A). We counted the number of peaks in peak-related genes and found that the number of peaks on most genes was 1 (Supplementary Figure S1B). Compared with the NC group, 867 specific m^6^A peaks were found in the RSA group. This indicated that there was a significant difference between the RSA group and NC group in the overall m^6^A modification patterns. Next, statistical analysis was performed between the two groups to find the m^6^A peaks with statistically significant differences. One hundred and one m^6^A modification peaks (genes) were up-regulated significantly and 37 peaks (genes) were down-regulated significantly in the RSA group (fold change >2, *p* < 0.05) and the volcano plot was conducted (Figure 1B).

It has been reported that m^6^A methylation modulators can regulate the m^6^A modification by recognizing specific sequences which are also called consensus motifs: RRACH (R for A or G, H for A, T, C) (Liu et al., 2014). To further understand the m^6^A modification in the RSA patients, the motifs of the m^6^A modification were studied. The results showed that the top consensus motifs identified m^6^A peaks were GGACT which were consistent with the previous studies (Figures 1C,D). We also found that m^6^A peaks were mainly enriched in the stop codon regions and 3′ untranslated regions (UTRs) (Figures 1E,F), which was consistent with the previous study that m^6^A modification sites preferentially map near stop codons and the 3’ UTRs (Meyer et al., 2012).

### M^6^A Modification May Be Related to RSA

Further analysis was performed on genes with significantly different m^6^A modifications between the two groups. The Gene Ontology (GO) analysis revealed that the genes with significantly different m^6^A modification in the RSA group were enriched in many pathways involved in biological processes related to growth and development, such as cell proliferation, biological adhesion, and reproduction (Figure 1G). KEGG analysis revealed that the transcripts with unique m^6^A peak in RSA were involved in the toll-like receptor (TLR) signaling pathway, natural killer cell–mediated cytotoxicity, Herpes simplex virus infection, necrotizing apoptosis, and hepatocellular carcinoma. (Figure 1H). Studies have shown that TLR was expressed in placental cells and participated in the initiation of signal mediators that were essential for the immune response in choriocarcinoma cell lines, which may be related to placental inflammation (Klaffenbach et al., 2005). Similarly, cytotoxicity mediated by natural killer cells (NK) can lead to endometritis, form a uterine immune state, and then lead to infertility, recurrent abortion, and so on (Chen et al., 2020; Li et al., 2020; Gu et al., 2021). Other studies have shown that apoptosis can affect the differentiation and invasion of trophoblast cells, block the process of vascular recasting, change the function of the placenta, and finally lead to embryo implantation, growth restriction, and abortion (Sharp et al., 2010). Therefore, the m^6^A modification may affect the function of trophoblast cells through the aforementioned biological functions and pathways. Also, m^6^A modification may be related to RSA.

### RNA Expression Profile Changes in the RSA Group and It Correlates With Disregulations in m^6^A Modifications

**FIGURE 2:**
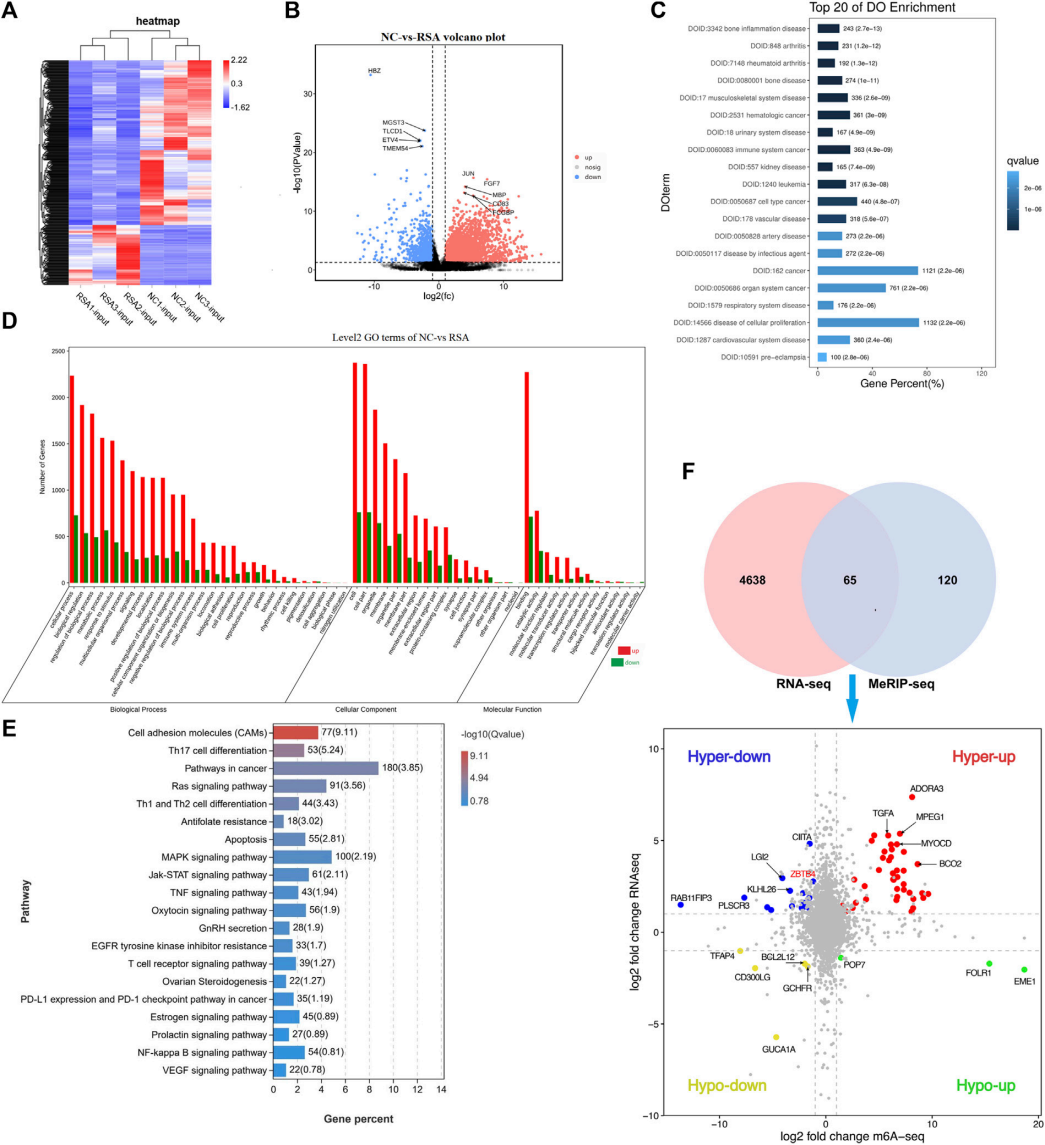
Overview of mRNA profile in the two groups and the correlation with m6A modification. **(A)** Heatmap conducted according to the RNA-seq data. In the heatmap, each column represented a sample, and each row represented a gene. The expression levels of genes in different samples are represented by different colors. The redder the color, the higher the expression level, and the bluer the color, the lower the expression level. **(B)** Volcano plot conducted according to the RNA-seq data. The volcano plot showed the differentially expressed genes between the two groups. The farther the genes were from the center of the graph, the greater the degree of difference in the expression between the two groups. FDR <0.05 and |log2FC| > 1 were considered with significantly different. **(C)** DO (Disease Ontology) histogram was conducted according to the differential expression genes and showed the top 20 items. Q value ≤0.05 was considered with significantly different. **(D)** GO histogram was conducted according to the differential expression genes. **(E)** KEGG pathway was conducted according to the differential expression genes showed 20 pathways that may be associated with RSA. **(F)** Venn diagram showed the genes with significant differences both in m^6^A modification and mRNA levels. Quadrant diagram showed the distribution of 65 significant differential genes.

In order to study the interaction between the m^6^A modification and the gene expression in RSA patients, we also conducted the RNA sequencing (RNA-Seq) in the RSA group and NC group. From the results of RNA-seq, we found that there was a significant difference between the RSA group and NC group in the gene expression patterns. Compared with the NC group, 4,703 mRNAs were present with significant differences, including 3,634 up-regulated mRNAs and 1,069 down-regulated mRNAs (FDR <0.05, |log2FC| > 1). The heatmap and volcano plot were conducted and visually showed the differences between the two groups (Figures 2A,B).

DO analysis was conducted and the top 20 items are shown in Figure 2C. We can see that the differential genes were significantly enriched in items related to reproductive system diseases, cellular proliferation diseases, vessel-related diseases, and cancer. GO analysis showed the differentially expressed genes significantly enriched in GO terms related to cell function regulation (cellular process, cell proliferation, cell killing, cell aggregation, development process, and immunity function regulation). Figure 2D. As far as we know, embryonic villous implantation disorder was one of the causes of RSA, which may be related to cell dysfunction such as cell adhesion, cell migration, cell motility, and cell activation. The KEGG analysis revealed the possible pathways related to RSA. The top items related to RSA, cell proliferation, and migration are Th17 cell differentiation, Th1 and Th2 cell differentiation, Ras signaling pathway, cancer-related pathway, antifolate, apoptosis, tumor necrosis factor signaling pathway, MAPK signaling pathway, oxytocin signaling pathway, gonadotropin secretion, T-cell receptor signaling pathway, PD-L1 expression and PD-1 checkpoint pathway in tumors, prolactin signaling pathway, and Ovarian Steroidogenesis (Figure 2E).

To clarify the correlation between m^6^A modification and the gene expression and to find the potential targets related to RSA, we performed a correlation analysis between the total expression profile and m^6^A modification profile (Supplementary Figure S2A). A total of 65 genes were found with significant differences both in m^6^A modification and mRNA expression (Figure 2F). According to the upward or downward trend of m^6^A modification and mRNA expression, 65 genes were divided into four categories (Figure 2F, Supplementary Figure S2B). The methylation modification of m^6^A has been proven to be reversible, and this process required the participation of methyltransferases (writers), demethylases (Erasers), and methylation readers (Readers). We have noticed that m^6^A methyltransferase METTL3 was significantly down-regulated in the RSA group. Therefore, the genes whose m^6^A modification decreased while the expression increased became the main genes of interest and 14 genes among the 65 genes were selected in the hyper-down quadrant. Based on the gene function, we finally selected six genes (EMILIN1, MTR, LGI2, ZBTB4, TFAP4, and PLSCR3) which were closely related to cell proliferation, migration, and apoptosis (Danussi et al., 2012; Khot et al., 2017).

### ZBTB4 Was One of the Targets of METTL3 by Regulating m^6^A Modification in Trophoblast

**FIGURE 3:**
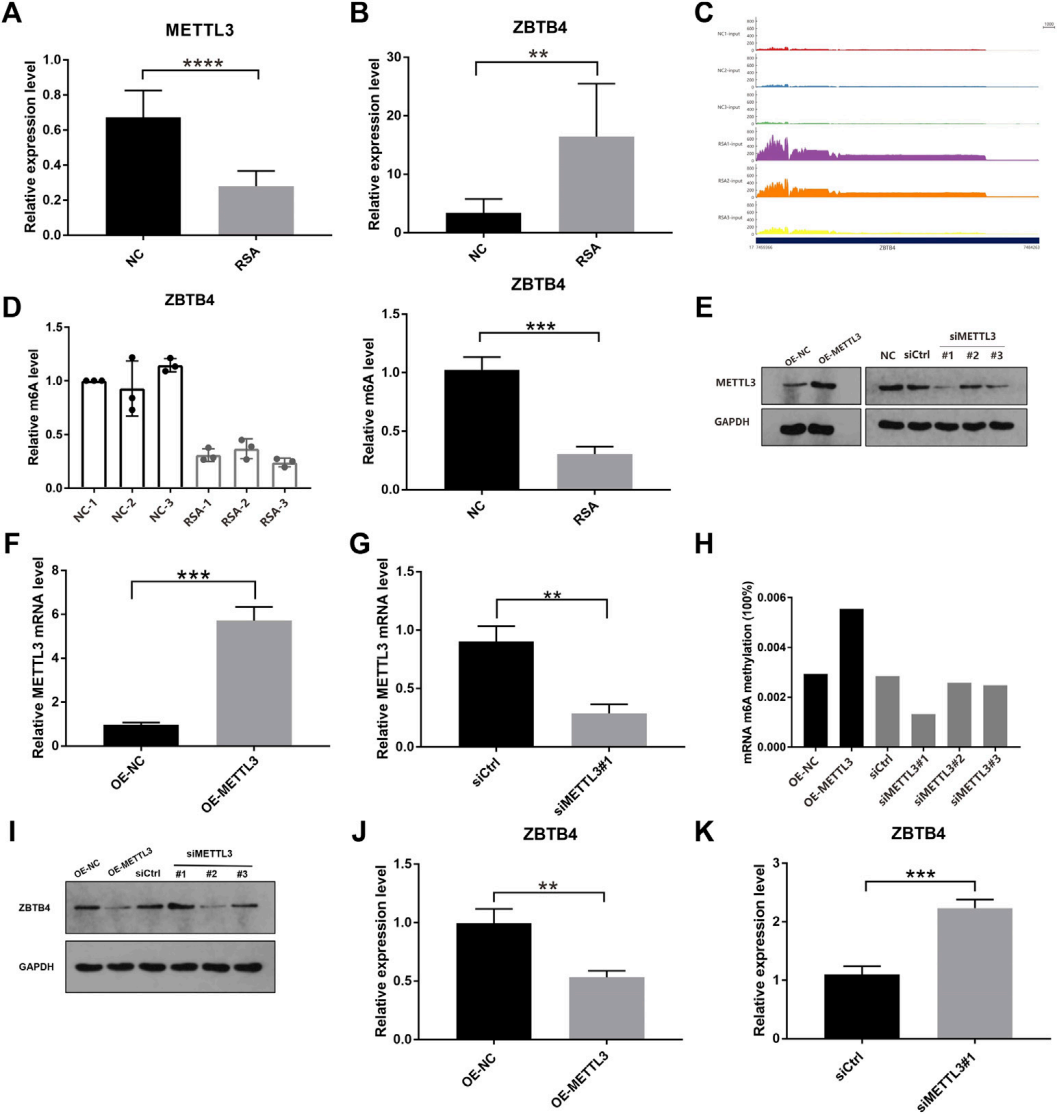
ZBTB4 was one of the targets of METTL3 by regulating m6A modification in trophoblast. **(A, B)** Relative mRNA levels of METTL3 and ZBTB4 were verified by using real-time PCR in RSA and control groups. **(C)** RNA-seq data shows that ZBTB4 mRNA was significantly up-regulated in RSAs compared with controls and the homogeneity of ZBTB4 mRNA level between samples was better. **(D)** Changes of the m^6^A levels of ZBTB4 in RSA and control samples were verified by gene-specific MeRIP-qPCR. **(E–G)** Western blotting and qRT-PCR analysis of METTL3 in HTR-8 cells with METTL3 overexpression or METTL3 knockdown. siMETTL3 oligonucleotide (#1) was the best one for METTL3 knockdown. **(H)** RNA m^6^A modification was assessed using the m^6^A RNA Methylation Quantification ELISA kit in HTR-8 cells with METTL3 overexpression or METTL3 knockdown. **(I–K)** Western blotting and qRT-PCR analysis of ZBTB4 in HTR-8 cells with METTL3 overexpression or METTL3 knockdown.

We further detected METTL3 mRNA expression levels in the RSA group and NC group by real-time quantitative PCR (RT-qPCR), and the results were still consistent with the results of RNA-Seq (Figure 3A). The decreased expression level of METTL3 might mean that the modification of m^6^A reduced in the RSA group. The mRNA expression levels of the selected six genes were also detected by RT-qPCR. Five genes out of six were consistent with the results of RNA-Seq. Among them, ZBTB4 was one of the most significant differentially expressed genes (Figure 3B). The RT-qPCR results of the remaining five genes are shown in Supplementary Table S1. From the results of RNA-seq, we found that the homogeneity of ZBTB4 mRNA between samples was good (Figure 3C). In addition, MeRIP–qPCR was conducted to test the level of ZBTB4 m^6^A modification in the RSA group and NC group. The level of ZBTB4 m^6^A modification was increased in the NC group and decreased in the RSA group, which was consistent with the result of MeRIP-Seq (Figure 3D). Previous studies reported that ZBTB4 was a transcriptional inhibitor that inhibited cell proliferation and metastasis (Roussel-Gervais et al., 2017). Therefore, we hypothesized that the decrease in the METTL3 expression in RSA patients may lead to the down regulation of m^6^A modification abundance of the ZBTB4 gene, which may increase the expression of ZBTB4, and finally affect the implantation of villous cells and lead to the occurrence of RSA.

In order to further explore whether ZBTB4 was the substrate of METTL3 and the role of METTL3 in human trophoblast, the HTR-8/SVneo (HTR-8) cell line was selected for the knockdown and overexpression of METTL3 using the METTL3 expressing vector and three siMETTL3 oligonucleotides, respectively. In the HTR-8 cells with the METTL3 overexpression, the METTL3 expression was increased. In the HTR-8cells with METTL3 knockdown, the METTL3 expression was down-regulated and the first siMETTL3 oligonucleotide (#1) was the best one for METTL3 knockdown (Figures 3E–G). To understand the effect of METTL3 on RNA m^6^A modification, we carried out the m^6^A quantitative experiments. From the results, we can see that the global level of m^6^A of the METTL3 knockdown HTR-8 cells (siRNA#1) was decreased, while the global level was increased in the METTL3 overexpressing HTR-8 cells (Figure 3H). These results showed that METTL3 could regulate the level of m^6^A modification through its own expression. In addition, the overexpression of METTL3 significantly down-regulated the expression of ZBTB4 in HTR-8 cells, and the knockdown of METTL3 significantly up-regulated the ZBTB4 expression (Figures 3I–K). In summary, these results showed that ZBTB4 was one of the targets of METTL3 which down-regulated the ZBTB4 expression through adjusting m^6^A modification abundance.

### METTL3 Regulated the Expression of ZBTB4 by Recognizing ZBTB4 mRNA m^6^A Motifs in the CDS

**FIGURE 4:**
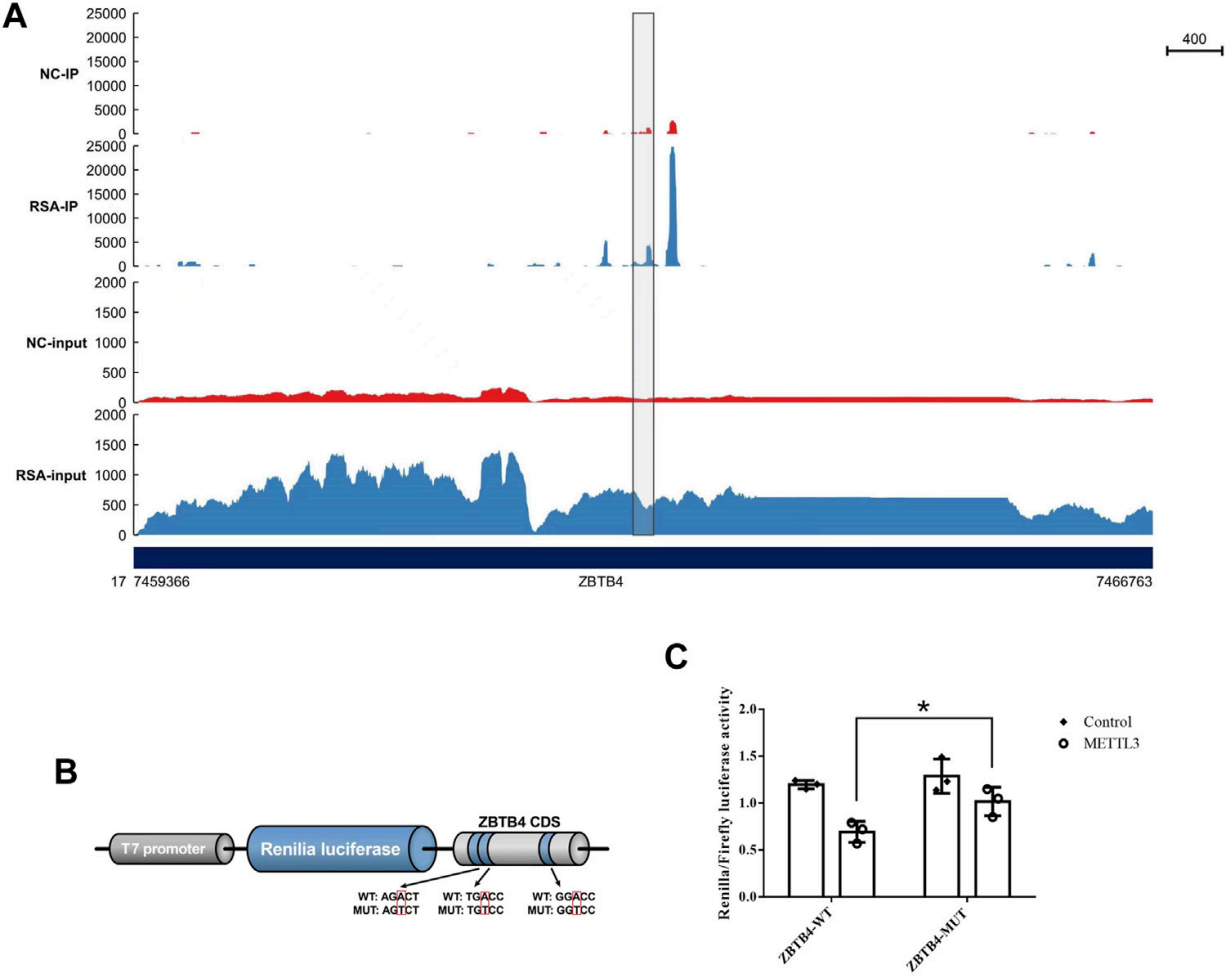
METTL3 mediated m^6^A modification of m6A motifs in ZBTB4 CDS. **(A)** m^6^A peaks in ZBTB4 mRNA transcripts in RSA and control samples as detected by MeRIP-seq. The m^6^A peaks with a significant increased or reduced abundance (fold change >2, *p* < 0.05) are shown in the grey frame. **(B)** Wild-type and mutant fragments of ZBTB4 CDS were cloned into the downstream of the Renilla luciferase gene of psiCheck2 vector. **(C)** Renilla luciferase activity was detected and normalized to that of the firefly luciferase. Three independent experiments were conducted.

In order to study whether METTL3 regulated the expression of ZBTB4 by recognizing ZBTB4 mRNA m^6^A motifs in CDS, we conducted the dual-luciferase reporter assay. The ZBTB4 CDS containing m^6^A motifs (Figures 4A,B) was inserted into the downstream of the dual-luciferase vector and the luciferase activity in transfected cells was detected. The luciferase activity was significantly reduced in the cells transfected with the METTL3 overexpression plasmid (Figure 4C). These results showed that METTL3 mediated the ZBTB4 expression by acting on the ZBTB4 CDS.

### METTL3 Regulated the Invasion of Trophoblast by Altering the Stability and Expression of ZBTB4

**FIGURE 5:**
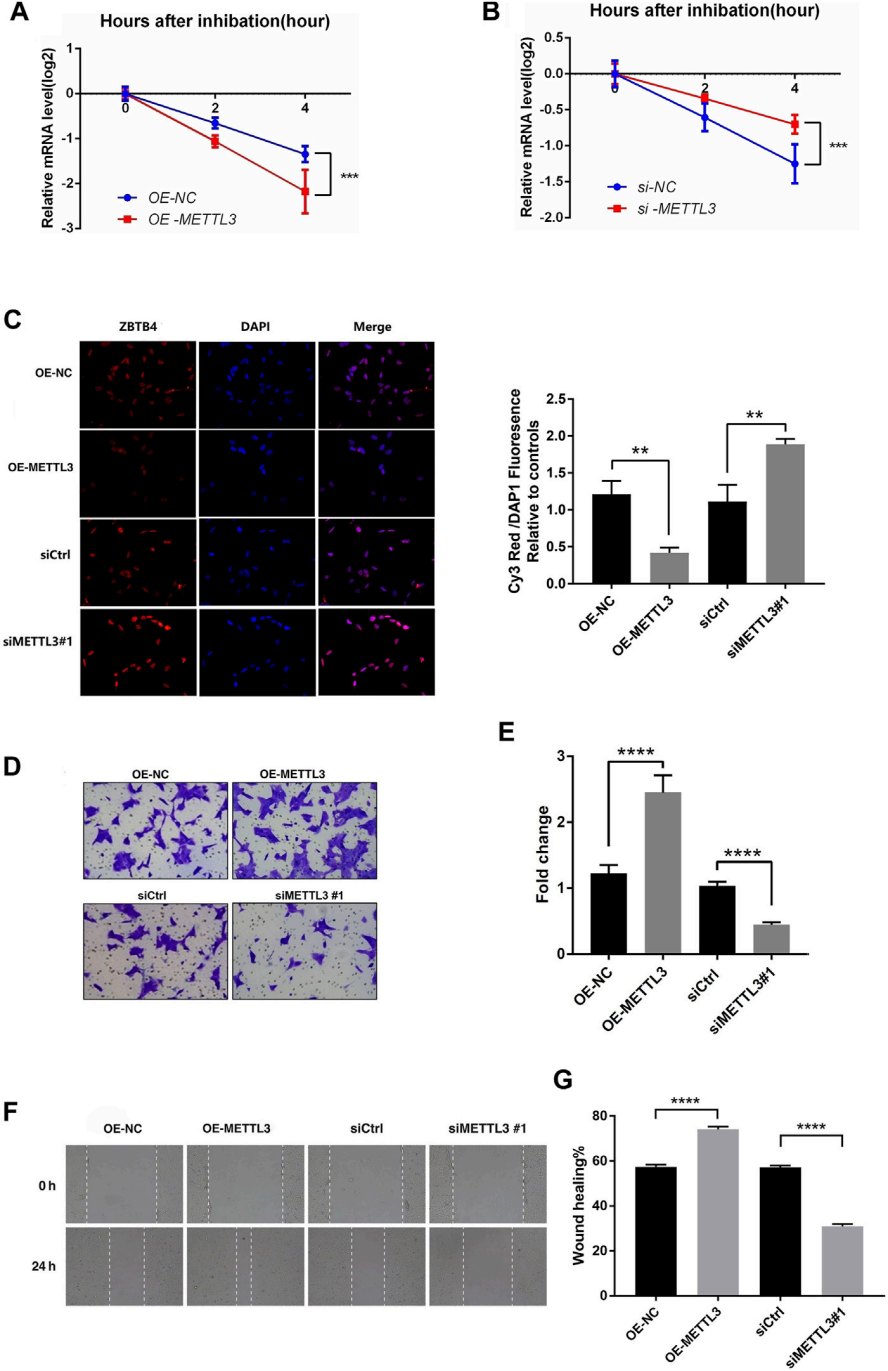
METTL3 regulated the invasion of trophoblast by altering the stability and expression of ZBTB4. **(A,B)** HTR-8 cells were transfected with siCtrl, siMETTL3, vector or METTL3-expressing plasmid for 24 h; a time course for mRNA stability was initiated by adding an RNA-polymerase II inhibitor [actinomycin D (5 μg/ml)]. Cells were harvested at the indicated time points. Expression levels were normalized to that at “0 h,” and GAPDH mRNA was used as the reference gene. The results are shown as the mean of at least three independent experiments. *p < 0.05 versus the vector or siCtrl. **(C)** Results of immunofluorescence. Magnification = ×400. **(D,E)** METTL3 overexpression in HTR-8 cells increased trophoblast invasion compared to that of the control. The invasive ability of the cells was assessed using Image-Pro Plus 6.0 software. (Panel: original magnification ×100 magnification). **(F,G)** Images were taken over time at 0 and 24 h. White dashed lines delineate the wound area. Magnification = ×40.

It was known that m^6^A modification can affect the stability, splicing, and expression of mRNA, thereby affecting a variety of cell biological functions. According to the results of our study, we can see that the m^6^A modification of ZBTB4 was regulated by METTL3. In order to explore whether m^6^A modification affect the stability of ZBTB4 mRNA, we treated the HTR-8 cells (transfection of siCtrl, siMETTL3 #1, and METTL3-overexpressing plasmids with RNA-polymerase II inhibitor actinomycin D (5 μg/ml) and detected the ZBTB4 mRNA level at 0, 2, and 4 h after actinomycin D treatment. The results showed that METTL3 had a strong effect on the stability of ZBTB4 mRNA. Compared with the control group, the half-life of ZBTB4 mRNA was increased after transfection with siMETTL3 oligonucleotide. On the contrary, the half-life of ZBTB4 was significantly reduced after METTL3 overexpressing treatment (Figures 5A,B). These results showed that METTL3 regulated the expression of ZBTB4 may be by affecting the stability of mRNA.

In order to illustrate the role of METTL3-mediated m^6^A modification in RSA trophoblast cells, we selected the HTR-8 cells transfected with siMETTL3 (#1) and overexpressed METTL3 for matrigel invasion assays and wound healing assays. According to the previous results, after transfection with the METTL3 overexpression vector, the METTL3 expression was increased, while the ZBTB4 expression and stability were decreased. After siMETTL3 transfection, the METTL3 expression was decreased, while the ZBTB4 expression and stability were increased. Immunofluorescence assays were conducted to verify the expression of ZBTB4 after transfection with the METTL3 overexpression vector and siMETTL3 transfection. The results of immunofluorescence are shown in Figure 5C. The results were consistent with the previous assays. The matrigel invasion assays showed that overexpression of METTL3 significantly promoted the invasion ability of HTR-8 cells, while interference of the METTL3 expression significantly inhibited the invasion of HTR-8 cells (Figures 5D,E). The wound healing assays got the same result as matrigel invasion assays (Figures 5F,G).

It was known that ZBTB4 was a transcription inhibitor that can inhibit the gene expression and reduce the invasion ability of cells. It can be speculated that interference of METTL3 expression in HTR-8 cells can enhance the stability and expression of ZBTB4, thus inhibiting the proliferation and invasion of HTR-8 cells. Overexpressing METTL3 had the opposite result. To sum up, METTL3 may regulate the invasion of trophoblast by altering the stability and expression of ZBTB4 mRNA.

## Discussion

The present study indicated that there was a novel mechanism of proliferation and migration associated with m^6^A methylation in RSA trophoblast cells. The reduction of METTL3-mediated m^6^A modification weakened ZBTB4 attenuation and increased ZBTB4 expression, which weakened the invasion ability of trophoblast. This may lead to the inadequate implantation of early embryos and impaired trophoblast cell vessel recasting, and result in poor pregnancy outcomes.

As one of the epigenetic regulation, m^6^A methylation was involved in the RNA-associated metabolism process, such as RNA transcription, processing, splicing, degradation, and translation (Zhao and Cui, 2019). In recent years, research studies have shown that many genes were related to the occurrence and development of a variety of cancers due to abnormal m^6^A methylation modification, such as renal clear cell carcinoma, breast cancer, glioblastoma, liver cancer, prostate cancer, and so on. It also potentially affected the development of embryos as reported (Zhao et al., 2017; Kasowitz et al., 2018; Xiao et al., 2019). METTL3 was one of the key m^6^A methyltransferase which contained METTL3, WTAP, METTL14, and KIAA1429 (Roundtree et al., 2017). M^6^A methyltransferase was involved in the post-transcriptional methylation of adenosine residues in the mRNA of eukaryotic cells to form N6-methyladenosine (Zhang et al., 2021). In addition, studies have shown that METTL3 can directly act on the translation initiation machinery and promote m^6^A modification of mRNA, regardless of the activity of methyltransferase and m^6^A reader. Therefore, METTL3 can not only methylate mRNA and recognize methylated mRNA, but also directly affect the translation of mRNA (Lin et al., 2016). Currently, researchers are showing more interest in the potential roles and mechanisms of METTL3 in various biological processes. It has been reported that METTL3 knockdown can delay the development of embryos after the 4-cell stage and further lead to embryo development defects, which may be related to the increase of mis-localization of hnRNPA2/B1 and the decrease of m^6^A RNA methylation (Kwon et al., 2019). In addition, studies have shown that m^6^A RNA modification catalyzed by METTL3 was important for the integrity of IAP heterochromatin in mouse embryonic stem cells (Xu et al., 2021). It has also been reported that zebrafish embryos with WTAP and METTL3 knocked out show a variety of developmental defects, suggesting that WTAP and METTL3 can affect embryonic tissue differentiation (Fang et al., 2019). However, the underlying mechanism of METTL3 affecting embryonic development was still unclear. In this study, we found that m^6^A modification in RSA trophoblast cells was significantly different from that in the normal control group through MeRIP-seq, and the pathways of the differential m^6^A modified genes enriched have been reported to be related to the pathogenesis of RSA such as toll-like receptor signaling pathway, natural killer cell–mediated cytotoxicity, and necrotizing apoptosis. (Klaffenbach et al., 2005; Sharp et al., 2010; Chen et al., 2020; Li et al., 2020; Gu et al., 2021). The authors pointed out that URSA patients, compared with normal pregnant women, were enriched in several signaling pathways associated with immune regulatory functions, including natural killer cell–mediated cytotoxicity, cytokine receptor interaction, and other signaling pathways which were consistent with our findings (Klaffenbach et al., 2005; Sharp et al., 2010; Chen et al., 2020; Li et al., 2020; Gu et al., 2021).

In addition, to clarify the correlation between m^6^A modification and the gene expression and to find the potential targets related to RSA, we performed RNA-seq. A correlation analysis between the results of MeRIP-seq and RNA-seq was performed. We found that the METTL3 expression was significantly decreased in RSA, while the ZBTB4 expression was significantly increased. Through RT-PCR verification experiments, gene function analysis, etc., we speculated that ZBTB4 was one of targets of METTL3 and related to the RSA by regulating the m^6^A modification and expression of ZBTB4 in trophoblast.

It was well known that the placenta was indispensable for successful pregnancy and normal fetal development. As a temporary exchange organ, the placental trophoblast cell line differentiates into an extravillous trophoblast (EVT), whose migration and invasion of trophoblast cells at the maternal–fetal interface was a key process of embryo implantation. Inadequate trophoblast invasion or failure to reshape the uterus and blood vessels can lead to common obstetric complications such as miscarriage, premature delivery, fetal growth restriction, fetal death, and preeclampsia (Kaufmann et al., 2003; Soares et al., 2014). A recent study on the global landscape of m^6^A modification of fetal tissues showed that the degree of differential m^6^A modification in placental tissue was the highest, followed by muscle tissue, suggesting that m^6^A modification may be implicated in some of the placental diseases (Xiao et al., 2019). ZBTB4, a mammalian DNA-binding protein, was widely recognized as a transcriptional inhibitor and played an indispensable role in the genesis, development, and metastasis of cancer (Yu et al., 2018). In our study, we first found that the ZBTB4 expression was significantly increased and the METTL3 expression was significantly decreased in RSA trophoblast tissue. To further clarify whether the expression alteration of ZBTB4 was related to METTL3-mediated m^6^A modification, we conducted a series of cell experiments using the HTR-8 cell line. The novel findings of this study included: 1) Modification mode of m^6^A methylation in RSA was changed; 2) The expression of m^6^A methyltransferase METTL3 was decreased in RSA, whereas the ZBTB4 expression was increased; 3) Through the knockdown and overexpression of METTL3 experiments using the HTR-8/SVneo (HTR-8) cell line, we found that ZBTB4 may be one of the targets of METTL3; 4) Through the dual-luciferase reporter assay, we found that METTL3 regulated the expression of ZBTB4 by recognizing ZBTB4 mRNA m^6^A motifs in the CDS; 5) Through the matrigel invasion assay, we found METTL3 regulated the invasion of trophoblast at the maternal–fetal interface by altering the stability and expression of ZBTB4. These results indicated that m^6^A methyltransferase METTL3 may play a key regulatory role in the proliferation and migration of RSA trophoblast cells.

In summary, our study revealed the mechanism by which METTL3 regulated the stability and expression of ZBTB4 and the trophoblast migration ability of RSA, and further explored the occurrence and development of RSA from the perspective of epigenetics. Most of the research on METTL3 and ZBTB4 focuses on their impact on the occurrence and development of tumors. Our research has extensively studied the various roles of METTL3 and ZBTB4 in human diseases, and reported for the first time that the interaction of METTL3 and ZBTB4 may be involved in regulating the function of placental trophoblast cells and affecting embryo implantation and development. This study laid a foundation for further research on the epigenetic change mechanism of the occurrence and development of RSA, and provided a new scientific basis for the clinical prevention and treatment of RSA.

## Data Availability

The datasets presented in this study can be found in online repositories. The names of the repository/repositories and accession number(s) can be found below: https://www.ncbi.nlm.nih.gov/bioproject/, PRJNA818161.
