# Supplementary material for: METTL3-Mediated m6A RNA Methylation of ZBTB4 Interferes With Trophoblast Invasion and Maybe Involved in RSA
Source: Front Cell Dev Biol. 2022 Jun 14;10:894810. doi: 10.3389/fcell.2022.894810 (PMC9237410; doi:10.3389/fcell.2022.894810)
Supplement: Supplementary file 1 [file Table1.docx]

Table S1 The RT-qPCR results of the remaining 5 genes

| Gene Name | group | Mean CT of the targe gene | Mean CT of β-Actin | P Value | Regulation (NC VS RSA) |
| --- | --- | --- | --- | --- | --- |
| MTR | NC | 24.59661844 | 17.2616682 | 0.0456 | up |
|  | RSA | 24.31406932 | 17.6371477 |  |  |
| TFAP4 | NC | 26.50019381 | 16.7501175 | 0.0056 | down |
|  | RSA | 27.49340322 | 16.6192202 |  |  |
| EMILIN1 | NC | 23.64699862 | 15.9021433 | 0.7837 | up |
|  | RSA | 25.25611941 | 17.2461349 |  |  |
| LGI2 | NC | 29.06623385 | 17.6707756 | 0.0214 | up |
|  | RSA | 28.7638002 | 18.3234749 |  |  |
| PLSCR3 | NC | 24.35348479 | 18.3167347 | 0.0047 | up |
|  | RSA | 23.90580675 | 18.5055812 |  |  |
