# Supplementary figures and images for: METTL3-Mediated m6A RNA Methylation of ZBTB4 Interferes With Trophoblast Invasion and Maybe Involved in RSA
Source: Front Cell Dev Biol. 2022 Jun 14;10:894810. doi: 10.3389/fcell.2022.894810 (PMC9237410; doi:10.3389/fcell.2022.894810)

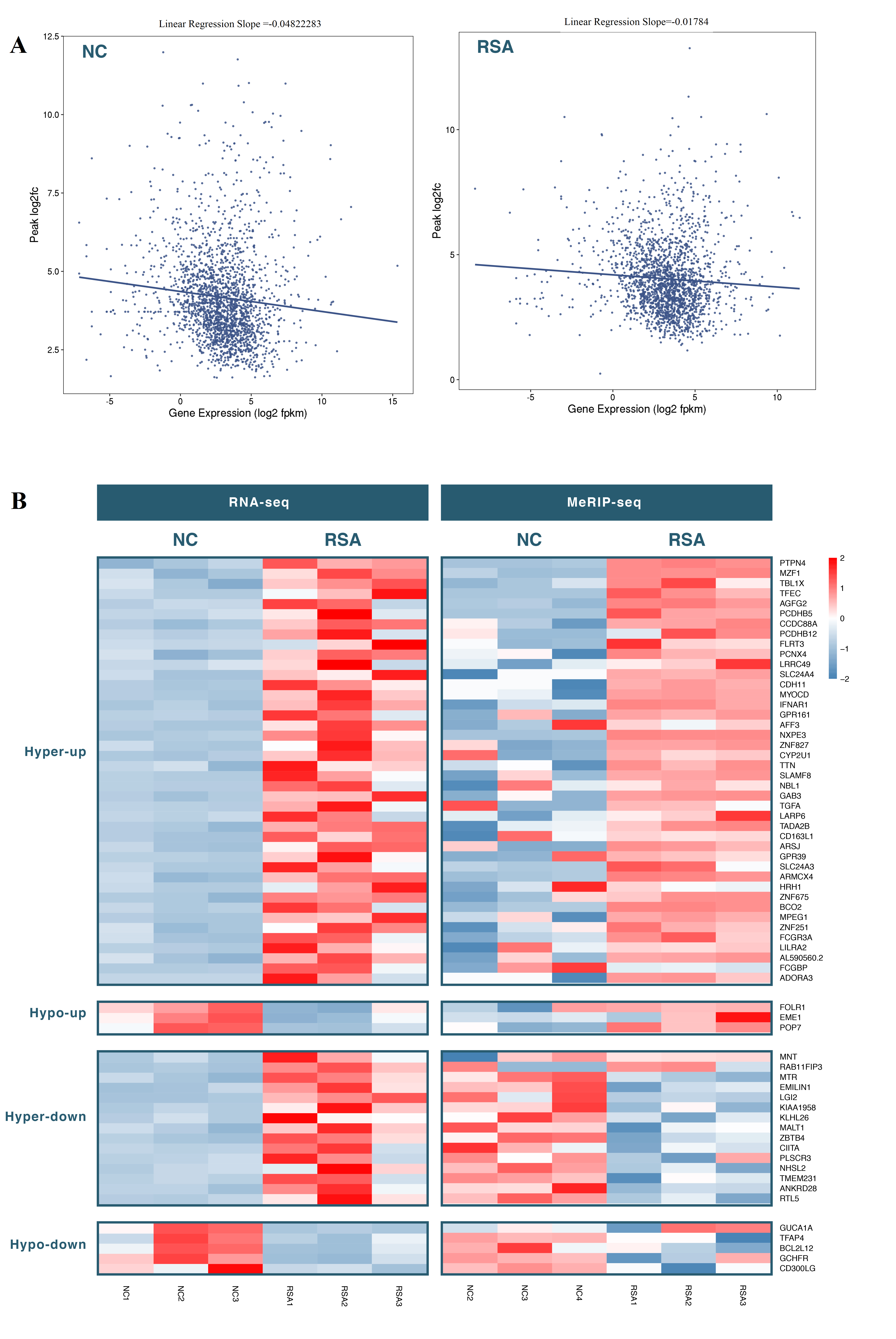

Supplement: Supplementary file 2 [file Image2.PNG]

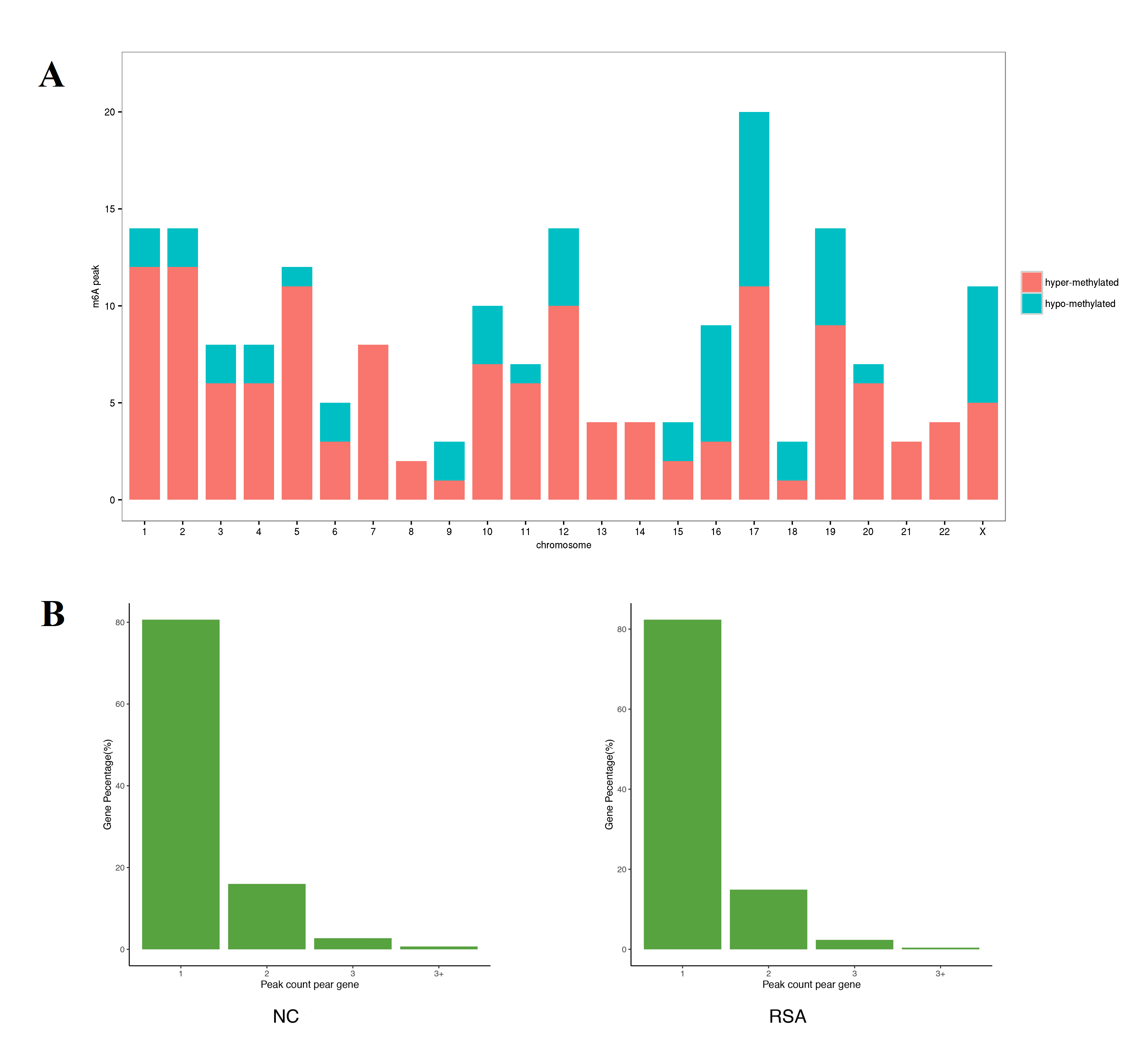

Supplement: Supplementary file 3 [file Image1.PNG]
